# Functional Gene Group Analysis Reveals a Role of Synaptic Heterotrimeric G Proteins in Cognitive Ability

**DOI:** 10.1016/j.ajhg.2009.12.006

**Published:** 2010-02-12

**Authors:** Dina Ruano, Gonçalo R. Abecasis, Beate Glaser, Esther S. Lips, L. Niels Cornelisse, Arthur P.H. de Jong, David M. Evans, George Davey Smith, Nicolas J. Timpson, August B. Smit, Peter Heutink, Matthijs Verhage, Danielle Posthuma

**Affiliations:** 1 Department of Functional Genomics, Neuroscience Campus Amsterdam VU University, 1081 HV Amsterdam, The Netherlands; 2 Department of Molecular & Cellular Neurobiology, Center for Neurogenomics and Cognitive Research, Neuroscience Campus Amsterdam VU University, 1081 HV Amsterdam, The Netherlands; 3 Department of Medical Genomics, VU Medical Center, Neuroscience Campus Amsterdam VU University, 1081 HV Amsterdam, The Netherlands; 4 MRC Centre for Causal Analyses in Translational Epidemiology, Department of Social Medicine, University of Bristol, Bristol BS8 2BN, UK; 5 Center for Statistical Genetics, Department of Biostatistics, University of Michigan, Ann Arbor, MI 48109, USA

## Abstract

Although cognitive ability is a highly heritable complex trait, only a few genes have been identified, explaining relatively low proportions of the observed trait variation. This implies that hundreds of genes of small effect may be of importance for cognitive ability. We applied an innovative method in which we tested for the effect of groups of genes defined according to cellular function (functional gene group analysis). Using an initial sample of 627 subjects, this functional gene group analysis detected that synaptic heterotrimeric guanine nucleotide binding proteins (G proteins) play an important role in cognitive ability (P_EMP_ = 1.9 × 10^−4^). The association with heterotrimeric G proteins was validated in an independent population sample of 1507 subjects. Heterotrimeric G proteins are central relay factors between the activation of plasma membrane receptors by extracellular ligands and the cellular responses that these induce, and they can be considered a point of convergence, or a “signaling bottleneck.” Although alterations in synaptic signaling processes may not be the exclusive explanation for the association of heterotrimeric G proteins with cognitive ability, such alterations may prominently affect the properties of neuronal networks in the brain in such a manner that impaired cognitive ability and lower intelligence are observed. The reported association of synaptic heterotrimeric G proteins with cognitive ability clearly points to a new direction in the study of the genetic basis of cognitive ability.

## Introduction

Cognitive ability is a highly heritable trait, with heritability estimates ranging from 40% in young childhood to 80% in late adulthood.^1^, ^2^, ^3^ Disturbance in cognitive functioning ultimately causes lower test intelligence and is related to various psychiatric conditions, including schizophrenia, mental retardation, and autism. Identifying the actual genetic (and environmental) factors influencing cognitive ability may therefore elucidate the etiological basis of individual differences in both normal and abnormal cognitive functioning.^4^ Typically, each of the reported DNA variants associated with cognitive ability explain less than 1%–2% of the variation, and common variants in only a few genes (cholinergic receptor, muscarinic 2, *CHRM2* [MIM 118493], catechol-O-methyltransferase, *COMT* [MIM 116790], and brain-derived neurotrophic factor, *BDNF* [MIM 113505]) have repeatedly shown associations.^5^ Despite the high heritability of cognitive ability and the major efforts made to understand it during the past decades, we are currently left with the generally accepted conclusion that “cognitive ability is most likely influenced by many genes of small effect, that possibly interact,”^6^, ^7^ without knowing the identity of these genes.

In the past decade, the scale of genotyping and genetic association studies has increased rapidly, from single-locus analysis to genome-wide association studies (GWAS) that allow the screening of 500,000–1,000,000 SNPs, covering 65%–95% of the human genome, depending on the array of choice. This has proven a successful method of identifying common genes of relatively large effect^8^, ^9^, ^10^, ^11^, ^12^, ^13^ but has been less effective when rare variants of large effect are of importance or when identifying genes of small effect for complex traits, such as attention-deficit hyperactivity disorder (ADHD [MIM 143465]) or schizophrenia (MIM 181500).^14^, ^15^

**Figure 1:**
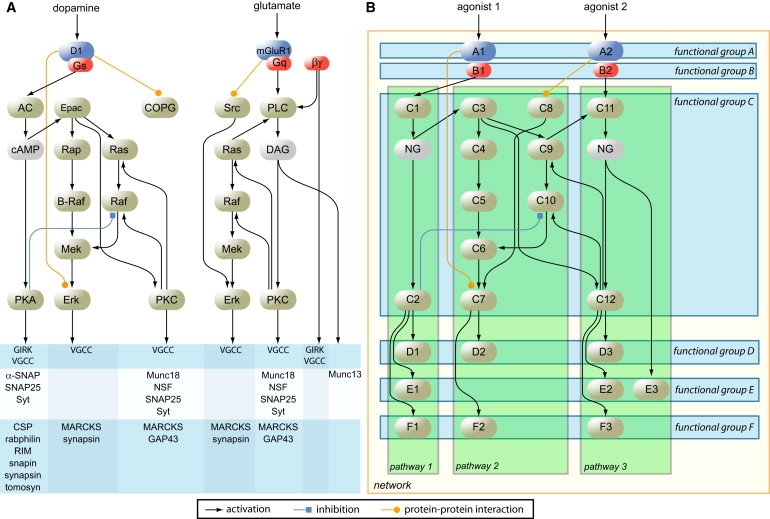
Schematic Representation of Vertical Pathways versus Horizontal Functional Groups (A) Genes that are involved in two different biological pathways (actual dopamine and glutamate pathways) are illustrated. It can be clearly seen that genes are not exclusively active in one pathway, but tend to be part of more than one pathway. (B) Genes are grouped according to similar cellular function, and naturally each gene is exclusively assigned to one functional group.

Collective testing of genes involved in biological pathways has emerged as an alternative strategy for testing the combined effects of genetic variants with small effect size.^16^, ^17^ Such pathways are usually defined as a set of proteins that participate in cascades of intracellular reactions, often triggered by extracellular ligands, involving enzyme-catalyzed posttranslational modifications of proteins and/or changes in their subcellular distribution. This ultimately leads to changes in cellular responses, such as altered gene expression or adaptation of cell morphology. Indeed, testing the combined effect of multiple genetic variants in such pathways has been shown to be more powerful than testing single-gene effects.^17^ Many proteins, however, are known to act *across* pathways, suggesting that they are not exclusively linked to one pathway. For instance, different neuromodulators, such as dopamine or 5HT, activate receptors in the plasma membrane, which are functionally and often structurally similar to the receptors in other pathways. A higher degree of convergence exists for downstream signaling steps, such as guanine nucleotide binding proteins (G proteins), effector enzymes, second messengers, kinases, and classes of substrates. It is conceivable that genetic variation that influences complex traits accumulates at foci of convergence that act *across* different pathways—but have similar cellular function—and that can influence multiple biological pathways in a similar manner. We refer to grouping genes according to cellular function as “horizontal grouping,” as opposed to the classical grouping of genes in pathways, which we refer to as “vertical grouping” (Figure 1).

Horizontal grouping may be especially powerful in *synaptic* protein networks. Many different pathways have been described, all of which modulate synaptic function. Importantly, these pathways have a high degree of convergence, which has a common consequence; i.e., many modulate synaptic strength and change the coupling between neurons. Therefore, it is conceivable that genetic variation in (many) different pathways leads to similar consequences in synaptic function and hence to a common phenotypic effect. Collective testing of genes *across* pathways addresses this possibility.

Previous gene-finding studies for cognitive ability have focused on targeted candidate-gene testing or whole-genome linkage or associations studies. Here, we focus on evaluating the combined effect of multiple genes in both vertical pathways and horizontal functional gene groups. Results from the functional gene group approach clearly suggest the involvement of synaptic heterotrimeric G proteins in cognitive ability.

## Subjects and Methods

### Study Subjects

Subjects were part of the international collaborative research effort on the genetics of ADHD (the International Multi-Center ADHD Gene project [IMAGE]), and data of these subjects were obtained from the Database of Genotypes and Phenotypes (dbGaP). The IMAGE project was one of the six projects selected by the National Institutes of Health (NIH) in 2006 to be genotyped in the first phase of the Genetic Association Information Network (GAIN).

Characterization of the samples used in the IMAGE project has been described elsewhere in detail.^18^, ^19^, ^20^ In brief, 947 nuclear families of European descent (2844 individuals) from eight countries (Belgium, England, Germany, Holland, Ireland, Israel, Spain, and Switzerland) were included in the analysis. In this sample, standardized intelligence quotient (IQ) scores were available for 627 unrelated subjects (87.7% males). The age at the time of data collection for these 627 subjects ranged from 5 to 19 yrs, with a mean age of 11.0 (standard deviation [SD] = 2.7). Their IQ was assessed with the Wechsler Intelligence Scale for Children (WISC-IIIR),^21^ in accordance with procedures described by Sattler.^22^ No information on medication status or treatment was included in the analysis.

### Genotyping and Quality Control

Genotyping was performed by Perlegen Sciences with the use of their genotyping platform, which comprises 600,000 tagging SNPs designed to be in high linkage disequilibrium with untyped SNPs for the three HapMap populations. Genotype data were cleaned by the National Center for Biotechnology Information (NCBI) with the use of the GAIN QA/QC software package before being uploaded to the dbGaP database. Details of the quality control (QC) process have been reported elsewhere.^23^ A total of 438,783 SNPs survived QC and were available in the downloaded data set.

### Definition of Functional Gene Groups and Biological Pathways

As opposed to defining groups of genes according to the neuromodulator involved, we grouped genes horizontally; that is, across the classically defined pathways. Functional gene grouping was based on cellular function and relies on previous protein identification and data mining for synaptic genes and gene function. Because no selective purification method exists for the purification and analysis of presynaptic proteins, the criterion for proteins from the presynapse was based on manually curated data mining and confirmed by comparison to proteomic data from whole-synapse (pre plus post) analyses and previously characterized presynaptic subcomplexes in solubilized preparations.

Determination of genes expressed in the presynaptic terminal was based on the following set of criteria:

• Null mutation produces a secretion phenotype in synapses.
• Activation of the gene product (e.g., receptor) or blockade thereof modulates secretion in synapses.
• Immuno-electronmicroscopy detects the gene product in the terminal.
• The gene product is enriched in affinity-purified synaptic vesicle fraction.
• Overexpression produces a secretion phenotype in synapses.
• Immunocytochemistry colocalizes the gene product specifically with the presynaptic marker.
• The gene product is enriched in purified brain synaptosomes.
• Null mutation produces a secretion phenotype in secretory cells and the gene is expressed in the human brain.

Inclusion of postsynaptic proteins was based on the presence of proteins in proteomics analyses of the synapse, including preparations of synaptic membrane fractions^24^ as well as the postsynaptic density.^25^, ^26^

Synaptic genes are subdivided into 17 functional groups of genes on the basis of shared function into a biological process (i.e., horizontal grouping) and manually curated published data. These groups are as follows: cell adhesion and transsynaptic signaling molecules; cell metabolism (synaptic metabolic enzymes and their cofactors, excluding mitochondrial proteins); endocytosis (proteins involved in endocytosis); excitability (voltage-gated ion channels); exocytosis (proteins involved in regulated secretion); G protein relay (G protein subunits); GPCR signaling (G protein-coupled receptors); intracellular signal transduction (enzymes downstream of G protein or tyrosine kinase [TK] signaling); intracellular trafficking (vesicle adaptors, sorting proteins, motor proteins); ion balance/ transport (ion and solute carriers and exchangers); ligand-gated ion-channel signaling; neurotransmitter metabolism (metabolizing enzymes); peptide/neurotrophin signaling (neuropeptide, trophic factors, hormones); protein clustering (scaffolding proteins); RNA and protein synthesis, folding, and breakdown; structural plasticity (cytoskeletal proteins and their regulators); and TK signaling (tyrosine receptor kinases).

One group was formed of remaining genes that are known to be expressed in the synapse but do not share any cellular function with other genes (this gene group was called “unknown”).

In addition, four canonical pathways (vertical grouping) for synaptic modulation were defined: the dopaminergic, glutamatergic, serotonergic, and cannabinoid pathways. These pathway definitions are in line with previously published definitions and can be obtained, for example, from regular textbooks or online biological-pathway definitions. All locus IDs in each functional gene group and canonical pathway, as well as the main references used to determine the functional groups, are available online in Tables S1 and S2.

### Data Processing

**Figure 2:**
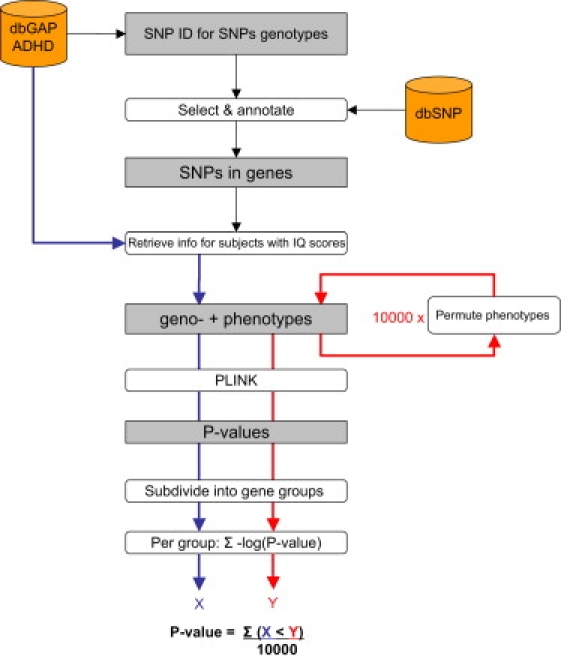
Data-Processing Steps From the dbGAP database, IDs were retrieved for SNPs genotyped in the GAIN-IMAGE study that included IQ phenotypes. From the dbSNP database, those SNPs that occur within and in the vicinity of genes were selected and annotated with the corresponding gene IDs. IQ scores were retrieved from the dbGAP database, as well as genotypes for the selected SNPs. p values were calculated for each SNP with PLINK. The SNPs and their p values were subdivided into functional groups. Per group, the negative log of all p values was summed to obtain value X. The last three steps were repeated after permutation of the IQ scores between subjects, giving a value Y for each permutation and each group. Per group, the number of times that Y was bigger than X was divided by the number of permutations to obtain the group's p value. Grey boxes represent data. Rounded boxes represent data-processing steps. Cylinders represent databases.

A schematic overview of the sequence of data-processing steps applied in this study is provided in Figure 2.

#### SNP Annotation

All SNPs that survived QC in the GAIN-IMAGE project were annotated according to the criteria of NCBI's SNP database (dbSNP). All SNPs that occur within or in the vicinity of genes were identified. A SNP was assigned to a gene on the basis of positions of the gene's reference sequence (RefSeq), with the addition of 2 kb to the 5′ end and 500 bp to the 3′ end of the gene. This region has been shown to include promoters (between −300 and −50 bp from the transcriptional start site [TSS]), as well as putative negative elements (between −1000 and −500 upstream of the TSS) and canonical hexanucleotide signals (20–50 bp from the 3′ end of the pre-RNA).^27^ SNPs mapped to multiple genes were assigned to each of these genes.

#### Statistical Analysis Method for Collective Testing of the Association of Gene Groups or Pathways

First, SNP-by-SNP analysis was carried out via linear regression. The Σ-log_10_(P) was calculated with the use of all p values obtained by single SNP analysis of selected SNPs in a group of genes, as defined above. The Σ-log_10_(P) is, however, not directly interpretable, because SNPs with the lowest p values will have the largest contribution to the calculated sum. As a result, this procedure can detect association of gene groups with a relatively low number of SNPs with low p values, even if some SNPs in the group have conventionally nonsignificant p values. In addition, the number of SNPs in a group will affect the test statistic Σ-log_10_(P), in the sense that a group containing more SNPs will have a higher Σ-log10(P) as compared to a group with fewer SNPs, assuming similar p values per SNP. Also, groups that contain SNPs with low p values and multiple SNPs in high linkage disequilibrium (LD) with those SNPs will have a higher Σ-log_10_(P) than will groups that contain SNPs of such low p values but without multiple SNPs in LD with those SNPs, given that p values are a function of LD between SNPs. Sample size will also affect the Σ-log_10_(P), because larger sample sizes are more likely to yield lower p values and thus higher Σ-log_10_(P).

So that unbiased interpretation of the test statistic was allowed, permutations (n = 10,000, or 100,000 when necessary) were carried out, conditional upon these factors, by permutation of phenotypes (i.e., the IQ scores) over genotypes. With this permutation procedure, only the relation between any genetic variant and the phenotype is disconnected, whereas the genomic haplotypic structure is kept intact. In addition, each selected group of genes will include exactly the same number of SNPs and genes, as well as the same haplotypic structure, and is based on the same sample size as that of the original data set.

For each group, we then determined by permutation how likely the observed Σ-log_10_(P) of that group was, given the number of SNPs and genes, the LD structure, and the sample size. A conservative Bonferroni correction was used to correct for multiple testing of different gene groups. Because we tested 23 different gene groups (all synaptic genes + 17 functional gene groups + one miscellaneous group and four biological pathways), the significance level was set at 0.05/23 = 0.0022. Given this signifcance level, the discovery sample of 627 subjects had sufficient power for detection of effect sizes of at least 2.4% of the total variation.

### Software

All SNP-by-SNP analyses were conducted in PLINK.^28^ SNP selection, permutation of the data sets, and calculations of the combined effect were implemented in scripts written in the R software package.

## Results

### IQ Scores Normally Distributed in the GAIN-ADHD Sample

**Figure 3:**
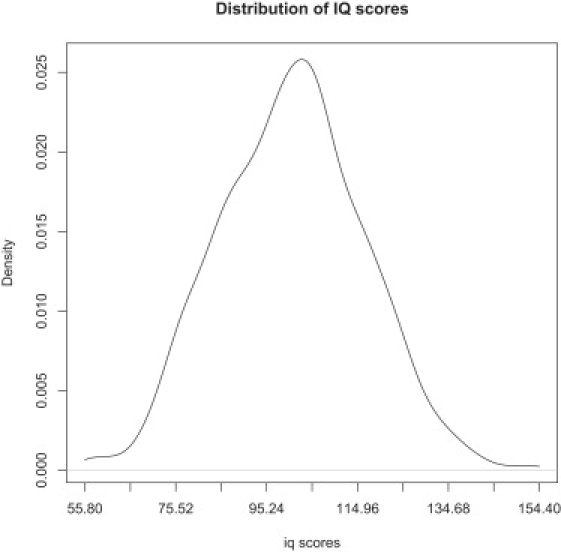
Distribution of the IQ Scores in the 627 Individuals Analyzed in the GAIN-IMAGE Sample

Phenotypic and genotypic data were obtained from dbGAP. IQ scores from 627 individuals were quantified with the Wechsler Intelligence Scales for Children (WISC)^29^ and had a mean of 100.7 (SD = 15.7) and a median of 101.6. Skewness was calculated to be 0.063, and the excess kurtosis relative to a normal distribution was −0.057 (Figure 3). Although this sample was originally ascertained for ADHD, this suggests that IQ scores are normally distributed in this sample.

### Genome-wide SNP-by-SNP Analysis Does Not Detect Robust Associations

**Figure 4:**
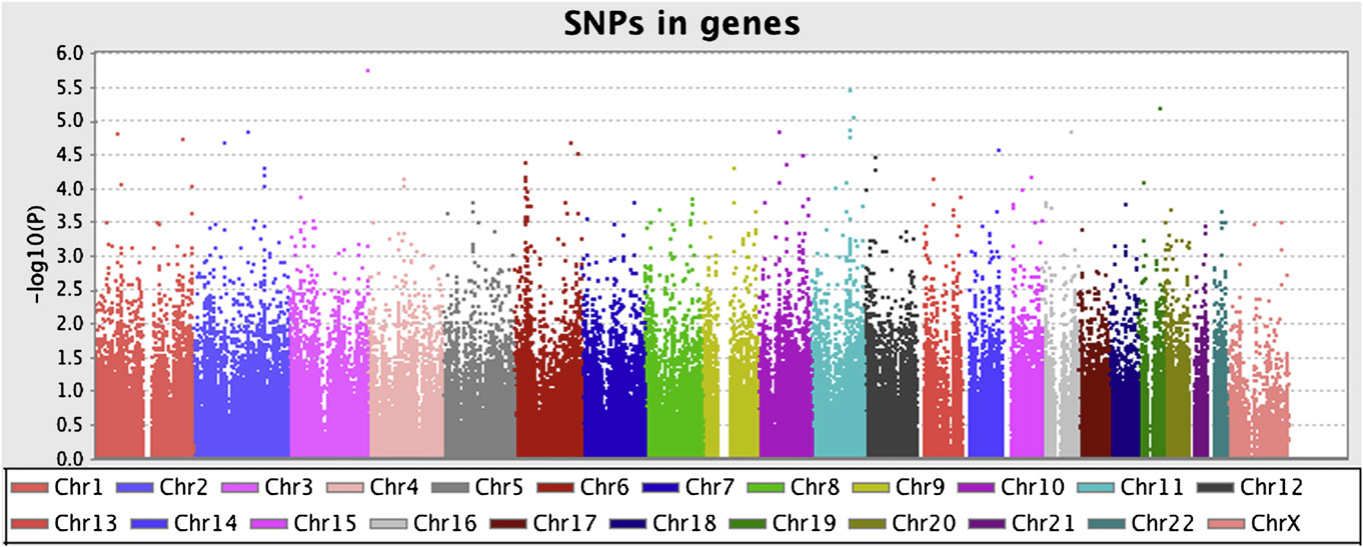
Manhattan Plot Showing Results of SNP-by-SNP Association with Cognitive Ability of the 179,725 SNPs Expressed in Genes Of these SNPs, 10,237 (5.7%) had p values below 0.05. At lower thresholds, the numbers of p values are as follows: 2230 lower than 1 × 10^−2^, 272 lower than 1 × 10^−3^, 38 lower than 1 × 10^−4^, 4 lower than 1 × 10^−5^, and the lowest p value was 1.8 × 10^−6^.

After QC, a total number of 438,783 SNPs were available for genome-wide association analysis. Of these, 179,725 SNPs mapped to 16,674 genes. The median number of SNPs per gene was 4.0, and the mean was 11.1, ranging from 1 to 1271 SNPs. First, we tested whether variation in single genes showed robust association with cognitive ability by applying a standard (SNP-by-SNP) genome-wide association analysis with the use of regression analysis implemented in PLINK^28^ and including only SNPs mapped to genes. Results of the SNP-by-SNP association analyses for all SNPs mapped to genes are shown in Figure 4.

With the genome-wide threshold being 1 × 10^−8^, none of the single SNPs reached significance. Obviously, this demonstrates mainly a lack of power and underscores the need for larger sample sizes or more sophisticated use of available information. With the current sample size of 627 subjects and a genome-wide significance level of 1 × 10^−8^, there was sufficient power for the detection of SNPs explaining at least 6.7% of the variance (Genetic Power Calculator^30^). For the detection of SNPs of small effect size (e.g., 2%) with reasonable power (0.80), a sample of 2138 subjects would have been needed.

### Analysis of Synaptic Functional Gene Groups Identifies Association with Cognitive Ability

**Figure 5:**
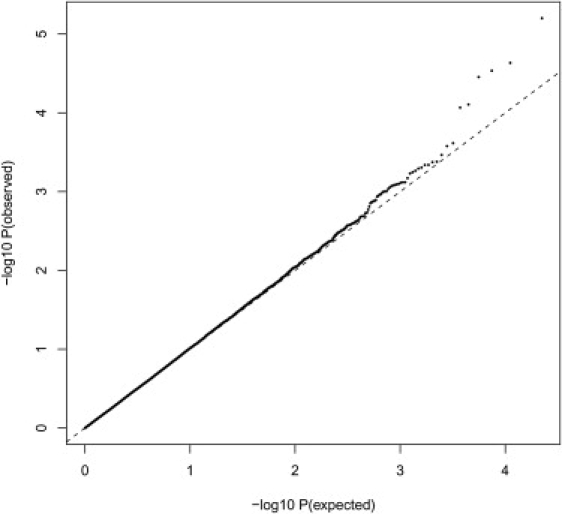
Results of Association Analysis of All Synaptic Genes with Cognitive Ability Distribution of expected (under the null hypothesis) versus observed p values for SNPs in genes expressed in synapses. The dashed diagonal line represents the line obtained if the observed distribution did not deviate from the expected distribution. All p values are corrected for λ.

Before testing detailed functional groups, we first tested whether genes expressed in synapses are associated with cognitive ability. This group contains 1024 genes, of which 900 (22,324 SNPs) were included on the Perlegen chip. Our results suggest that SNPs associated with cognitive ability are not randomly distributed across the genome, but tend to cluster in genes that are known to be expressed in synapses (p = 0.001) (Figure 5).

The 900 genes that are known to be expressed in the synapse and were included in the Perlegen chip were assigned to one of 17 functional gene groups on the basis of cellular function. Because some of the synaptic genes did not fit into any of the functional synaptic gene networks defined, we also tested this group of remaining synaptic genes as a negative control (the “unknown” group). The 49 genes in this group are expressed in the synaptic terminal, but their function is currently unknown.

**Figure 6:**
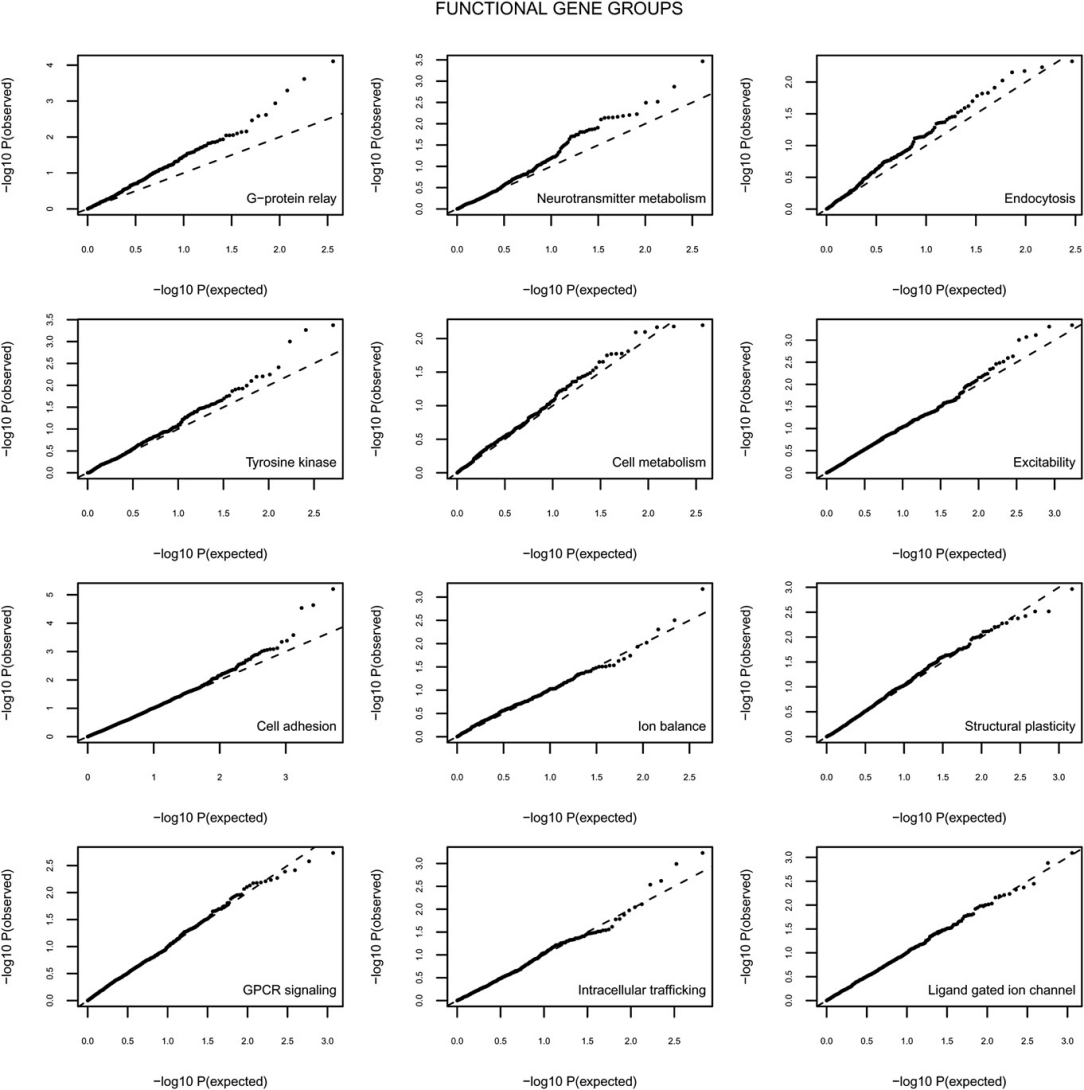
Results of Association Analysis Corrected for λ of the Twelve Most Significant Functional Gene Groups

**Figure 7:**
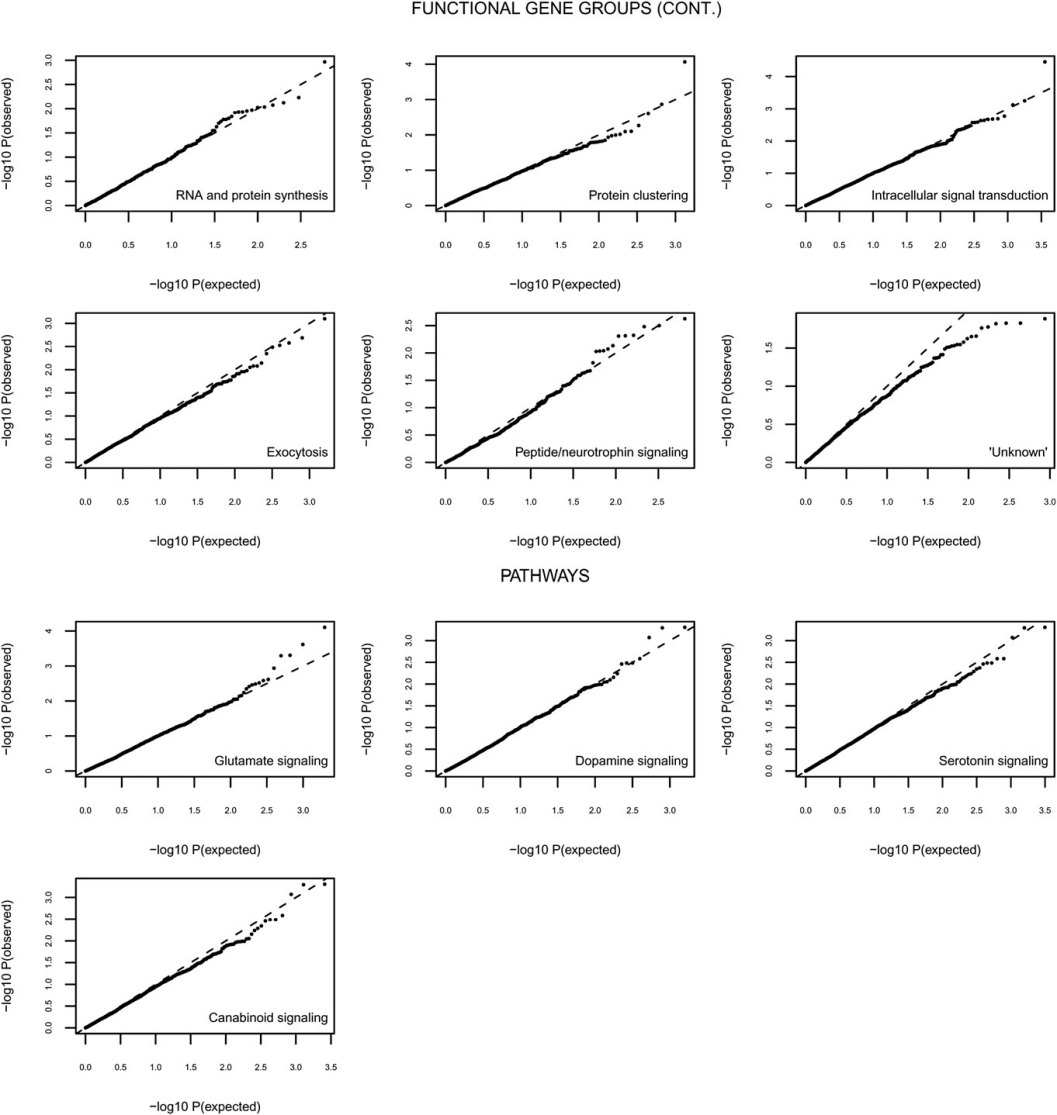
Results of Association Analysis Corrected for λ of the Remaining Functional Gene Groups and All Tested Biological Pathways

Table 1 lists the results of the joint association analysis of SNPs within each of the functional groups, and Figure 6 and Figure 7 provide the quantile-quantile (Q-Q) plots for each of the tested functional gene groups.

**Table 1:** Results of the Association Analysis of Functional Gene Groups and Biological Pathways with Cognitive Ability

**Gene Group**	**N Genes**	**N SNPs**	**Σ-log_10_(P)**	**P_EMP_**
All synaptic genes	900	22325	10146	**0.001**

**Synaptic Functional Gene Group**				
G protein relay	25	359	227	**0.00019**
Neurotransmitter metabolism	26	405	210	0.024
Endocytosis	24	291	153	0.058
Tyrosine kinase signaling	7	512	256	0.064
Cell metabolism	48	368	179	0.081
Excitability	48	1697	793	0.083
Cell adhesion and transsynaptic signaling molecules	74	5209	2359	0.109
Ion balance/transport	37	436	209	0.149
Structural plasticity	81	1474	676	0.160
GPCR signaling	37	1174	540	0.191
Intracellular signal transduction	137	3570	1591	0.236
Ligand gated ion channel signaling	36	1139	517	0.242
RNA and protein synthesis, folding and breakdown	58	597	266	0.341
Protein clustering	44	1322	582	0.395
Intracellular trafficking	66	667	293	0.406
Exocytosis	79	1593	681	0.603
Peptide/neurotrophin signaling	24	645	265	0.698
Unknown	49	867	347	0.880

**Biological Synaptic Signaling Pathways**				

Glutamate	60	1968	865	0.3883
Dopamine	69	1584	687	0.5006
Serotonin	102	3146	1348	0.6211
Cannabinoid	81	2568	1069	0.8309

Bold values indicate significance after correction for multiple testing. P***_EMP_*** denotes empirical p value, based on 10,000 (or 100,000 when necessary) permutations of the data. The groups have been ordered by their p values. For each selected group, the number of genes and the number of SNPs are listed. In the last two columns, the sum of the negative log_10_ of the p value in the original data and the empirical p value are listed for each group.

As expected, the “unknown” group did not show any evidence for an association with cognitive ability (empirical *P_EMP_* = 0.880). The group of synaptic heterotrimeric G proteins, however, yielded an overall test statistic for association with cognitive ability that was better than that expected for a group that size on the basis of chance alone (*P_EMP_* = 1.9 × 10^−4^), given the preset threshold of 0.0022. This suggests that the combined effect of genes within this group plays a role in explaining variation in cognitive ability. In the case of a single SNP, such a p value would correspond to an overall calculated effect size of at least 3.3% of the variation in cognitive ability.^30^ However, it should be noted that this calculated effect size (based on p value, sample size, and significance level) is based on single-SNP effects and is difficult to interpret when many (nonindependent) SNPs are involved.

None of the genes in the group of heterotrimeric G proteins have been associated with cognitive ability previously. This functional gene group consists of the following genes:

G protein alpha 11 (*GNA11* [MIM 139313]), G protein alpha 12 (*GNA12* [MIM 604394]), G protein alpha 13 (*GNA13* [MIM 604406]), G protein alpha 14 (*GNA14* [MIM 604397]), G protein alpha 15 (*GNA15* [MIM 139314]), G protein, alpha inhibiting activity polypeptide 1 (*GNAI1* [MIM 139310]), G protein, alpha inhibiting activity polypeptide 2 (*GNAI2* [MIM 139360]), G protein, alpha inhibiting activity polypeptide 3 (*GNAI3* [MIM 139370]), G protein, alpha activating activity polypeptide, olfactory type (*GNAL* [MIM 139312]), G protein, alpha activating activity polypeptide O (*GNAO1* [MIM 139311]), G protein, q polypeptide (*GNAQ* [MIM 600998]), GNAS complex locus (*GNAS* [MIM 139320]), G protein, alpha transducing activity polypeptide 1 (*GNAT1* [MIM 139330]), G protein, alpha z polypeptide (*GNAZ* [MIM 139160]), G protein, beta polypeptide 1 (*GNB1* [MIM 139380]), G protein, beta polypeptide 2 (*GNB2* [MIM 139390]), G protein, beta polypeptide 3 (*GNB3* [MIM 139130]), G protein, beta polypeptide 4 (*GNB4* [MIM 610863]), G protein, beta polypeptide 5 (*GNB5* [MIM 604447]), G protein, gamma 2 (*GNG2* [MIM 606981]), G protein, gamma 3 (*GNG3* [MIM 608941]), G protein, gamma 4 (*GNG4* [MIM 604388]), G protein, gamma 5 (*GNG5* [MIM 600874]), G protein, gamma 7 (*GNG7* [MIM 604430]), G protein, gamma 10 (*GNG10* [MIM 604389]), G protein, gamma 11 (*GNG11* [MIM 604390]), G protein, gamma 12 (*GNG12*).

Two genes in this group were not included in our analyses because there were no SNPs available in the Perlegen chip: *GNAT1* and *GNG3*. Notably, none of the single SNPs within this network would have been detected in the context of a genome-wide SNP-by-SNP analysis, with the lowest p value being 4.9 × 10^−5^. In fact, only four SNPs within this functional gene group reached a p value below 10^−3^, and only 12.8% of SNPs had a p value below 0.05. Three of these four SNPs with a p value below 10^−3^ are in the *GNAQ* gene, and the 46 SNPs with a p value lower than 0.05 are distributed throughout 11 different genes: *GNA14*, *GNAQ*, *GNG2*, *GNAS*, *GNG4*, *GNG11*, *GNB5*, *GNAI1*, *GNAL*, *GNAO1*, *GNB3*, with frequencies of 13, 9, 8, 5, 3, 3, 1, 1, 1, 1, and 1, respectively. This suggests that the effect of the functional group cannot be explained by the effect of a few individual SNPs or genes but must be ascribed to the combined effect of multiple genes in the functional gene group. This is also evident in the Q-Q plot of the heterotrimeric G proteins in Figure 6 (upper left panel), which shows that the distribution of the observed p values for the functional group of genes encoding heterotrimeric G proteins are consistently lower than expected under the null hypothesis of uniform distribution, with no single p value standing out.

### Biological Pathway Analysis Shows No Association with Cognitive Ability

Because the total collection of synaptic genes was associated with cognitive ability, we tested four biological pathways that are known to involve synaptic functioning as one of their roles in cognitive ability. These pathways involve the dopaminergic, glutamate, serotonergic, and cannabinoid pathways and follow the rationale of vertical grouping. Of these pathways, the serotonin pathway showed the largest number of genes overlapping with the synaptic genes. The dopaminergic, glutamatergic, serotonergic, and cannabinoid pathways yielded the following empirically derived p values: dopaminergic, *P_EMP_* = 0.5006; glutamatergic, *P_EMP_* = 0.3883; serotonergic, *P_EMP_* = 0.6211; cannabinoid, *P_EMP_* = 0.8309 (see also Table 1 and Figure 7), suggesting that collective testing of genes in synaptically relevant biological pathways is less successful in identifying genetic variation underlying cognitive ability than collectively testing genes that are grouped according to function in a biological process (horizontal grouping).

### Correction for Population Stratification

**Figure 8:**
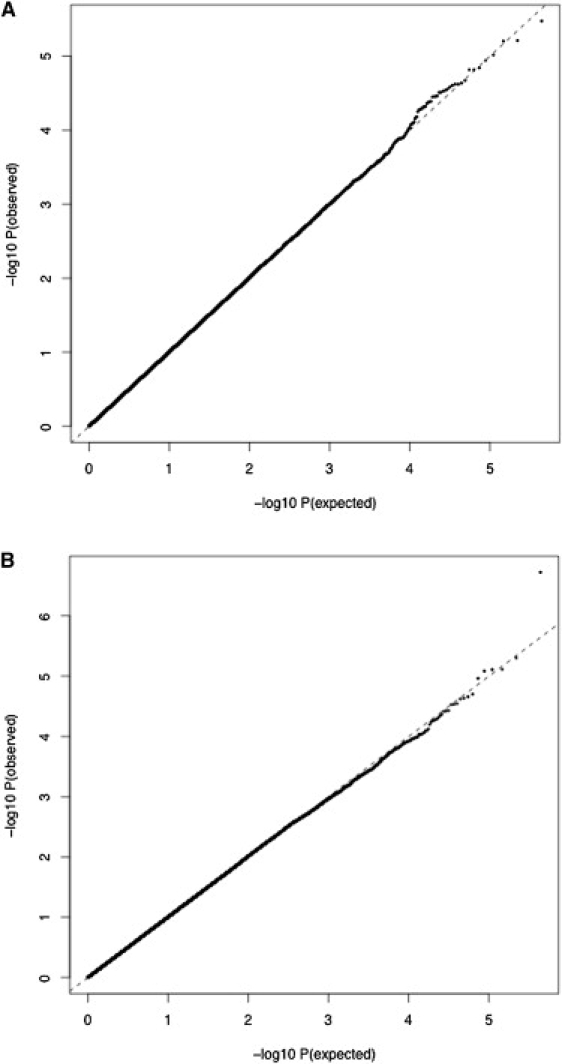
Distribution of Association Results for All SNPs that Survived QC Distribution of expected (under the null hypothesis) versus observed p values for all SNPs on the Perlegen platform that survived QC in the initial sample. The dashed diagonal represents the line obtained if the observed distribution did not deviate from the expected distribution. (A) Results of association with standardized IQ scores according to the IQ test manuals (all p values have been corrected for λ). (B) Standardized IQ scores additionally corrected for differences across collection sites.

The possible effects of population stratification on our results were explored via two methods. The Q-Q plot (corrected for λ) based on all SNPs available for analysis is provided in Figure 8A. Without correction for λ, a slight deviation from the uncorrected expected distribution of p values under the null hypothesis was present, which was quantified in a genomic inflation factor of λ = 1.05672. This deviation is may be due to true association or may be indicative of false positives due to population stratification. Applying the genomic control correction method, we corrected all test statistics by the genomic inflation factor. Although all synaptic genes were no longer statistically significant as a group, this did not significantly change the results for the heterotrimeric G proteins (*P_EMP_* = 0.00062).

Second, given that the primary sample is known to include subsamples as a result of data being collected in different sites and countries, we calculated *Z* scores within each site and conducted all analyses again. The *Z* score procedure ensures that there are no mean trait differences left across subpopulations and therefore rules out spurious associations due to the known subpopulation structure. The genomic inflation factor using the *Z* scores was calculated as λ = 1 (see Q-Q plot in Figure 8B). Again, the results remained significant (*P_EMP_* = 0.0015) for heterotrimeric G proteins.

On the basis of these results, we conclude that possible spurious effects due to population stratification cannot account for the detected association of the heterotrimeric G proteins and cognitive ability.

### Validation of Significant Functional Gene Group in Independent Population-Based Sample

The Avon Longitudinal Study of Parents and Children (ALSPAC) study served as a validation sample. ALSPAC is a UK-based, population-based, prospective birth cohort with extensive data collection on health and development of children and their parents, predominantly those of white European origin, and has been described previously.^31^ Ethical approval for the study was obtained from the ALSPAC Law and Ethics Committee and the local research ethics committees. Genotyping on 1543 individuals was initially performed with the Illumina HumanHap 300K BeadChip for 1568 blood-derived DNA samples. After QC, the clean data set comprised 1507 samples (excluding individuals with potential non-European ancestry, more than 5% missing genotype data, sex-inconsistent X-heterozygosity or a genome-wide heterozygosity of more than 36.4% or less than 34.3%).

Because this validation sample is based on a general population sample and does not consist of individuals ascertained on the basis of ADHD, any replicated effect would confirm that the observed association is related to cognitive ability in general and is not specific to individual differences in cognitive ability in an ADHD population. The validation sample (n = 1507) had 100% power for detection of an effect size of 3.3% against a significance level of 0.05 (one test conducted).

Cognitive ability was measured in children 8 yrs of age with the Wechsler Intelligence Scale for Children (WISC-IIIK).^29^ A short version of the test, consisting of alternate items only (with the exception of the coding task), was conducted by trained psychologists.^32^ Verbal (information, similarities, arithmetic, vocabulary, comprehension) and performance (picture completion, coding, picture arrangement, block design, object assembly) subscales were administered, the subtests scaled and scores for total IQ derived.

**Figure 9:**
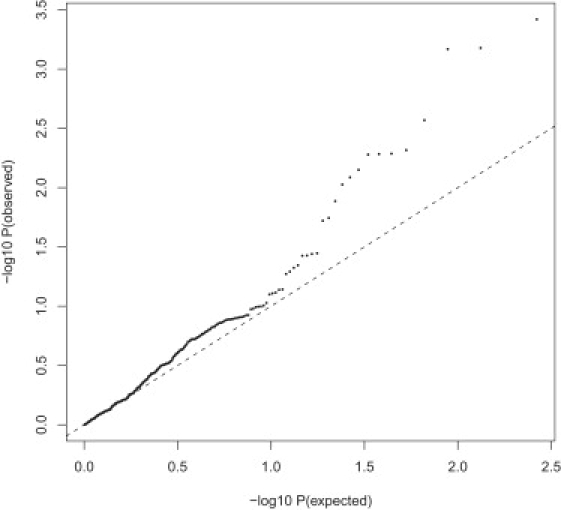
Q-Q Plot of All SNPs in the Heterotrimeric G Protein Relay Group in the Validation Sample

Of the 27 genes in the heterotrimeric G protein relay group, four genes were not covered in the validation sample. The validation sample included 265 SNPs mapped to 23 genes (versus 359 SNPs mapped to 25 genes in the original sample) in the G protein group. The difference in available SNPs was due to the difference in platforms used in the original and validation samples. The two genes (*GNB2* and *GNG11*) that were present in the original sample but were not covered in the validation sample include the *GNG11* gene, which was one of the most significant genes of the group, with three SNPs showing a p value < 0.05 (rs4262 = 0.009793; rs180236 = 0.01612; rs180241 = 0.02378). Figure 9 shows the Q-Q plot of all tested SNPs in the validation sample and suggests that all or most SNPs are important and that no single SNP drives the observed association of the G protein group.

Of the 46 SNPs in the G protein group that had a p value < 0.05 in the original data set, 27 SNPs had a proxy SNP with an r^2^ > 0.8, of which seven SNPs are identical between the two data sets and another seven SNPs have an r^2^ of 1. For reasons of comparison, gene coverage was determined on the basis of LD structure and genomic density and was based on the HapMap CEU LD structure. It was calculated by the sum of the typed SNPs as well as the tagged SNPs divided by the total known common SNPs within a gene (see Table 2).

**Table 2:** Gene Coverage Rates of the 27 Genes in the Heterotrimeric G Protein Group in the Initial and Validation Samples

		**HapMap (N SNPs)**		**Initial Sample (N SNPs)**			**Validation Sample (N SNPs)**		
**Gene**	**LID**	**Total**	**Common**^a^	**Genotyped**	**Tagged**^b^	**Cov. Rate**	**Genotyped**	**Tagged**^b^	**Cov. Rate**
*GNA11*	2767	29	24	3	0	0.13	7	9	0.67
*GNA12*	2768	180	118	21	87	0.92	16	82	0.83
*GNA13*	10672	57	8	2	5	0.88	2	5	0.88
*GNA14*	9630	348	217	73	68	0.65	39	82	0.56
*GNA15*	2769	29	20	7	3	0.50	6	4	0.50
*GNAI1*	2770	138	71	19	23	0.59	10	24	0.48
*GNAI2*	2771	14	3	1	0	0.33	1	0	0.33
*GNAI3*	2773	61	26	4	16	0.77	5	17	0.85
*GNAL*	2774	176	87	30	37	0.77	20	48	0.78
*GNAO1*	2775	242	143	29	60	0.62	20	84	0.73
*GNAQ*	2776	369	208	26	170	0.94	20	110	0.63
*GNAS*	2778	97	45	14	8	0.49	11	16	0.60
*GNAT1*	2779	3	1	0	0	0.00	0	0	0.00
*GNAZ*	2781	83	41	5	32	0.90	7	19	0.63
*GNB1*	2782	76	27	7	11	0.67	3	8	0.41
*GNB2*	2783	1	0	1	0	-	0	0	-
*GNB3*	2784	10	4	4	2	1.50	4	2	1.50
*GNB4*	59345	62	31	3	11	0.45	5	4	0.29
*GNB5*	10681	94	41	7	28	0.85	7	25	0.78
*GNG10*	2790	6	4	2	1	0.75	3	1	1.00
*GNG11*	2791	11	7	6	1	1.00	0	0	0.00
*GNG12*	55970	201	117	20	81	0.86	16	63	0.68
*GNG2*	54331	255	114	31	55	0.75	16	60	0.67
*GNG3*	2785	0	0	0	0	0.00	0	0	0.00
*GNG4*	2786	113	70	18	22	0.57	16	34	0.71
*GNG5*	2787	9	5	1	0	0.20	2	2	0.80
*GNG7*	2788	148	97	25	25	0.52	28	25	0.55

Total		2812	1529	359	746	0.72^c^	264	724	0.65^c^

Abbreviations are as follows: LID, locus ID; Cov. Rate, coverage rate.

a Common SNPs, based on MAF > 0.05.

b Tagged SNPs, based on pairwise r^2^ ≥ 0.80.

c Average overage rate.

In line with the recommendation by Holmans et al.,^16^ the validation study focused on the functional gene group rather than on a direct SNP-by-SNP replication. This may result in a conservative p value in the validation sample, given that the SNPs that showed most evidence for association in the initial sample are not directly included in the validation study. However, the main goal is to validate the association with the functional gene group, not with single SNPs. This is also in line with the assumption advocated here and in Holmans et al.,^16^ that it is more powerful to treat the functional gene group as the unit of analysis and not the single SNPs.

Analyses were conducted in PLINK^28^ similarly to the method applied in the original sample, and 10,000 permutations were used to determine the empirical p value of the combined effect of all included SNPs. The Σ-log_10_(P) of the heterotrimeric G proteins was 136, with an empirical p value of 0.047, validating our findings in an independent cohort.

## Discussion

Here, we applied an innovative functional gene group approach to cognitive ability. Functional gene groups were defined on the basis of shared cellular function of genes, as determined by previous protein identification and data mining for synaptic genes and gene function. Initially, we identified the group of synaptic heterotrimeric G proteins to be associated with cognitive ability in a relatively small sample of 627 subjects. We replicated these findings in an independent large cohort of 1507 subjects.

Association of the group of heterotrimeric G proteins could not be attributed to a single gene or a single SNP, because none of the individual p values came close to genome-wide significance, confirming the importance of focusing on the gene group as the unit of analysis and not on single-SNP effects.

Heterotrimeric G proteins consist of three subunits (α, β, and γ), for which a total of 33 genes are found in the human genome. Twenty-seven of these are ubiquitously expressed in the synapse. Many of these proteins are also expressed in the nonsynaptic areas of neurons and in other cells inside and outside the brain. Heterotrimeric G proteins are central relay factors between the activation of plasma membrane receptors by extracellular ligands and the cellular responses that these induce. Some signaling molecules in the brain can also activate ionic currents upon binding to ionotropic receptors, but for most signaling molecules no parallel or alternative pathways next to G protein receptor-coupled signaling exist to induce cellular responses. Therefore, heterotrimeric G proteins may be considered a point of convergence; a kind of “signaling bottleneck.” Although alterations in synaptic processes may not be the exclusive explanation for the association of heterotrimeric G proteins with cognitive ability, it is plausible that such alterations prominently affect the properties of neuronal networks in the brain in such a manner that impaired cognition and lower intelligence is observed. Synaptic processes are thought to have a central role in the “real time” processing capacities of the brain—for instance, in discrimination tasks, working memory, attention, and decision making^33^, ^34^, ^35^—as well as in adaptations required for “long-term synaptic modulation” in learning and memory.^36^

It is worth noting that the second most significant (although not below the conservative threshold of significance) functional gene group associated with cognitive ability in this study was the group of “transmitter synthesizing and metabolizing proteins,” which includes the *COMT* and monoamine oxidase A (*MAOA* [MIM 309850]) genes. Previous studies have systematically pointed to a role of metabolic enzymes in cognitive ability. In fact, the *COMT* gene has been associated with many different cognitive traits.^37^ These results suggest that further exploration of a role of the group of transmitter synthesizing and metabolizing proteins may be indicated.

### Evaluating the Combined Effect of Multiple Genes in Functional Gene Groups

The functional gene group approach may also prove fruitful for other complex traits or common disorders that are potentially influenced by many genes of small effect. One of the main assumptions of current GWAS is that common diseases are caused by at least a few common genetic variants of relatively large effect (the common disease, common variant [CDCV] hypothesis). If this assumption is not met (e.g., many common alleles with small effect, genetic heterogeneity), GWAS will not work, because in different individuals different variants will account for a disease status or trait level.^38^ However, if these different variants share a common molecular function, thus sustaining a common biological process, focusing on their combined effect will still be a valuable approach. The functional gene group approach may thus be able to detect genes even when there is large genetic heterogeneity at the SNP level, as long as the many alleles of small effect share a common function in a biological process.

Unlike previous methods, the functional gene group approach does not rely on post hoc formulation of pathways or networks, but instead takes a hypothesis-driven approach by directly testing functional gene groups. This is opposed to the commonly followed strategy in which the most significant SNPs from a genome-wide association analysis are annotated to search for possible biological pathways associated with the trait. Although this may prove a successful strategy, it will not detect any pathways or functional networks of genes in which most genes are of small and equal effect size, because it focuses initially on SNPs with the largest effects. The applied permutation procedure renders the functional gene group analysis independent of the number of SNPs per gene, of the number of genes per pathway or functional group, and of gene differences in LD structure. Functional gene group analysis may be further preferred because it circumvents the multiple-testing problem and because effect sizes are likely to be increased in comparison to single-SNP effects (because these are now a function of the combined effect, rather than of effects of single genes). This approach may therefore aid in resolving the “case of the missing heritability.”^15^ It should be noted, however, that the effect size as estimated in the current study explained 3.3% of the observed variation in cognitive ability, which is considered large in comparison to effects of single genes for cognitive ability but is still modest in terms of estimated heritability of cognitive ability. Additional pathways or functional gene groups are therefore also likely to contribute to variation in cognitive ability.

To our knowledge, this is the first study reporting on a functional role of synaptic heterotrimeric G proteins in cognitive ability, and it thereby directs future research into the genetic basis of cognitive ability toward synaptic signaling processes. At the same time, these results underscore the notion that pathway analysis or group analysis is more informative as the unit of analysis than is single-SNP analysis, because it is directly related to biological function. The functional gene group approach adapted in this study relies on grouping genes according to similar cellular function on the basis of extensive lab experiments (whole-synapse analyses and solubilized preparations) and data mining. This functional gene group approach complements existing methods^16^ and may also be useful in identifying etiological factors underlying complex diseases for which classic, genome-wide, SNP-by-SNP analysis has been unsuccessful so far.

## Web Resources

The URLs for data presented herein are as follows:

dbGaP, http://view.ncbi.nlm.nih.gov/dbgap-controlled
dbSNP, http://www.ncbi.nlm.nih.gov/SNP).
GAIN QA and QC software package http://www.sph.umich.edu/csg/abecasis/GainQC/
Genetics Cluster Computer, http://www.geneticcluster.org
Online Mendelian Inheritance in Man (OMIM), http://www.ncbi.nlm.nih.gov/Omim/
PLINK software, http://pngu.mgh.harvard.edu/∼purcell/plink/
R software, http://www.r-project.org

## Supplemental Data

Document S1. Two Tables
