# Supplementary material for: Functional Gene Group Analysis Reveals a Role of Synaptic Heterotrimeric G Proteins in Cognitive Ability
Source: Am J Hum Genet. 2010 Feb 12;86(2):113–25. doi: 10.1016/j.ajhg.2009.12.006 (PMC2820181; doi:10.1016/j.ajhg.2009.12.006)
Supplement: Document S1. Two Tables [file mmc1.pdf]

**Supplemental Data**
***AJHG*, Volume *86***

# Functional Gene-Group Analysis Reveals a Role of Synaptic Heterotrimeric G Proteins in Cognitive Ability

**D. Ruano, G.R. Abecasis, B. Glaser, E.S. Lips, L.N. Cornelisse, A.P.H. de Jong, D.M. Evans, G. Davey Smith, N.J. Timpson, A.B. Smit, P. Heutink, M. Verhage, and D. Posthuma**

TABLE OF CONTENTS

Table S1. Locus IDs of All Genes Included in the Synaptic Functional Gene Groups and Canonical Pathways

Table S2. Main References Used to Identify Functional Synaptic Groups and Canonical Pathways

Table S1. Locus IDs of All Genes Included in the Synaptic Functional Gene Groups and Canonical Pathways

| ***Functional gene groups*** |
|---|
| **EXCITABILITY**<br># genes: 59<br>LIDs: 773, 774, 775, 776, 777, 778, 781, 782, 784, 785, 3736, 3737, 3739, 3746, 3747, 3749, 3751, 3753, 3760, 3761, 3762, 3763, 3764, 3765, 3766, 3768, 3772, 3778, 3779, 6323, 6324, 6326, 6327, 6328, 6330, 6331, 6334, 6335, 7416, 7419, 7881, 8514, 9254, 9992, 10008, 10242, 10368, 10369, 23630, 23704, 27091, 27092, 27345, 55799, 55800, 57528, 59283, 93589, 115207 |
| **LiIGAND GATED ION SIGNALING**<br># genes: 36<br>LIDs: 1137, 1139, 1141, 2550, 2554, 2555, 2556, 2557, 2558, 2559, 2560, 2561, 2562, 2564, 2565, 2566, 2890, 2891, 2892, 2893, 2897, 2898, 2899, 2900, 2901, 2902, 2903, 2904, 2905, 2906, 3359, 8973, 9177, 9568, 11345, 348980 |
| **GPCR SIGNALLING**<br># genes: 41<br>LIDs: 134, 135, 148, 150, 154, 155, 577, 1128, 1129, 1268, 1269, 1394, 1395, 1812, 1813, 2911, 2913, 2914, 2915, 2917, 2918, 3350, 3351, 3352, 3356, 3357, 3363, 4985, 4986, 4988, 5023, 5024, 5027, 5028, 5029, 6000, 9127, 9256, 22859, 25927, 57512 |
| **G-PROTEIN RELAY**<br># genes: 27<br>LIDs: 2767, 2768, 2769, 2770, 2771, 2773, 2774, 2775, 2776, 2778, 2779, 2781, 2782, 2783, 2784, 2785, 2786, 2787, 2788, 2790, 2791, 9630, 10672, 10681, 54331, 55970, 59345 |
| **TYROSINE KINASE SIGNALING**<br># genes: 7<br>LIDs: 2043, 4915, 5786, 5793, 5799, 5802, 140885 |
| **INTRACELLULAR SIGNAL TRANSDUCTION**<br># genes: 150<br>LIDs: 107, 108, 109, 111, 114, 115, 387, 388, 472, 801, 805, 808, 810, 811, 814, 815, 816, 817, 818, 1152, 1267, 1457, 1460, 1495, 1496, 1499, 1500, 1501, 1612, 1620, 2029, 2885, 2932, 2977, 2982, 2983, 3094, 3208, 3241, 3265, 3631, 3706, 3845, 4082, 4884, 4900, 5037, 5048, 5049, 5121, 5138, 5245, 5331, 5332, 5335, 5337, 5500, 5516, 5518, 5524, 5530, 5532, 5533, 5534, 5566, 5567, 5573, 5575, 5576, 5577, 5578, 5579, 5580, 5581, 5582, 5590, 5594, 5595, 5604, 5780, 5879, 5898, 5908, 5910, 5911, 5912, 6093, 6261, 6262, 6263, 6500, 6620, 7070, 7447, 7529, 7531, 7532, 7533, 7534, 8437, 8536, 8613, 8936, 9201, 9454, 9456, 9475, 9479, 9732, 9922, 10314, 10409, 10458, 10486, 10487, 10857, 10971, 11069, 11113, 11261, 22808, 22933, 23096, 23162, 23236, 23362, 23467, 23542, 50488, 51062, 51440, 51517, 54332, 54769, 55450, 55512, 55915, 57026, 57118, 57172, 57447, 57596, 65108, 80728, 83988, 84446, 84687, 84894, 91860, 196883 |
| **STRUCTURAL PLASTICITY**<br># genes: 98<br>LIDs: 60, 71, 87, 118, 119, 120, 287, 288, 301, 302, 460, 830, 832, 998, 1072, 1428, 1465, 1627, 1780, 1808, 1809, 2036, 2037, 3796, 3800, 3837, 3925, 3927, 3983, 4130, 4131, 4133, 4135, 4137, 4627, 4628, 4637, 4741, 4744, 4747, 4763, 5064, 5216, 5217, 5339, 6624, 6709, 6710, 6711, 6712, 6950, 7114, 7280, 7430, |

| |
|---|
| 7846, 8522, 8976, 9118, 9211, 9253, 9362, 9890, 9948, 10006, 10092, 10093, 10094, 10096, 10097, 10152, 10381, 10382, 10383, 10391, 10398, 10529, 10552, 10570, 11034, 11151, 22924, 23122, 23136, 23406, 23499, 28988, 29114, 29767, 51286, 56896, 80086, 83660, 114299, 114787, 131034, 166336, 203068, 347733 |
| **NEUROTRANSMITTER METABOLISM**<br># genes: 29<br>LIDs: 1103, 1621, 2744, 4128, 4129, 6505, 6506, 6507, 6509, 6511, 6512, 6529, 6530, 6531, 6532, 6536, 6570, 6571, 6572, 7166, 9152, 23576, 26002, 57030, 57084, 121278, 140679, 246213, 388662 |
| **EXOCYTOSIS**<br># genes: 87<br>LIDs: 273, 378, 396, 2054, 4905, 5874, 6616, 6804, 6809, 6810, 6812, 6813, 6814, 6843, 6844, 6853, 6854, 6855, 6856, 6857, 6861, 8224, 8447, 8448, 8618, 8674, 8773, 8774, 8775, 9066, 9143, 9144, 9145, 9341, 9342, 9515, 9522, 9545, 9699, 9783, 9805, 9899, 9900, 10066, 10067, 10497, 10640, 10814, 10815, 11336, 22895, 22987, 22999, 23025, 23265, 23557, 25924, 29091, 54536, 54843, 55530, 55738, 55763, 55770, 55968, 60412, 63908, 80331, 80725, 84258, 84958, 91683, 94120, 94121, 94122, 112755, 116841, 127833, 132204, 134957, 143425, 148281, 149371, 192683, 201294, 252983, 440279 |
| **ENDOCYTOSIS**<br># genes: 26<br>LIDs: 160, 161, 162, 163, 1173, 1175, 1213, 1759, 1785, 6456, 8301, 8867, 8871, 8907, 8943, 9892, 10053, 10059, 10239, 10947, 22848, 25977, 26052, 28964, 51100, 56904 |
| **PEPTIDE/NEUROTROPHIN SIGNALING**<br>28<br>LIDs: 551, 627, 885, 1113, 1392, 1393, 2246, 3084, 4282, 4852, 4922, 5020, 5122, 5126, 5173, 5179, 5663, 5664, 6447, 6750, 7857, 9542, 10718, 23385, 51107, 55851, 83464, 145957 |
| **INTRACELLULAR TRAFFICKING**<br># genes: 79<br>LIDs: 392, 547, 1639, 1778, 2664, 2665, 3799, 3831, 3895, 4218, 4644, 5861, 5862, 5864, 5865, 5867, 5868, 5869, 5870, 5877, 5878, 6811, 6845, 6993, 7879, 8417, 8655, 8675, 9117, 9135, 9218, 9230, 9363, 9482, 10228, 10540, 10567, 10890, 11021, 11031, 11311, 22930, 22931, 23011, 23095, 23258, 23647, 23673, 24137, 25837, 26276, 27131, 27314, 27342, 30845, 51209, 51552, 51560, 51762, 53916, 53917, 55207, 55638, 57111, 57498, 64145, 64837, 65082, 79659, 79874, 81876, 84643, 89953, 115827, 116986, 140735, 143187, 201475, 376267 |
| **PROTEIN CLUSTERING**<br># genes: 47<br>LIDs: 989, 1487, 1488, 1739, 1740, 1741, 1742, 4355, 4356, 4735, 5413, 8495, 8496, 8497, 8499, 8500, 8541, 8573, 8825, 8927, 9228, 9229, 9463, 10580, 10801, 22839, 22866, 22884, 22941, 23085, 23157, 23176, 23349, 26059, 27445, 29993, 51678, 55752, 55964, 57513, 57524, 57554, 58512, 58538, 64130, 64398, 85358 |
| **CELL ADHESION AND TRANSSYNAPTIC SIGNALING**<br># genes: 81<br>LIDs: 214, 682, 708, 961, 999, 1000, 1001, 1002, 1003, 1004, 1005, 1006, 1007, 1008, 1009, 1010, 1012, 1013, 1014, 1016, 1272, 1400, 1404, 1463, 2017, 2064, 2817, 2823, 2824, 3897, 4045, 4062, 4155, 4340, 4345, 4684, 4685, 4756, 4974, 4978, 5067, 5097, 5354, 5818, 6091, 6092, 6900, 7087, 7143, 8502, 8506, 8642, 9369, 9378, 9379, 10675, 22871, 23114, 23705, 26047, 27020, 27255, 28316, |

| |
|---|
| 28513, 50863, 53942, 54413, 54798, 55914, 57502, 57555, 60437, 63827, 64072, 64405, 93185, 152330, 199731, 253559, 375790, 404037 |
| **ION BALANCE/TRANSPORT**<br># genes: 43<br>LIDs: 361, 476, 477, 478, 481, 482, 488, 490, 491, 492, 493, 523, 525, 526, 527, 528, 529, 534, 535, 537, 2697, 6521, 6543, 6546, 6547, 7779, 7781, 7782, 8671, 9114, 9296, 9368, 10396, 10463, 51382, 51606, 55676, 57282, 57348, 57468, 64924, 80727, 148867 |
| **RNA AND PROTEIN SYNTHESIS, FOLDING AND BREAKDOWN**<br># genes: 71<br>LIDs: 726, 821, 908, 1471, 1804, 1915, 1917, 1938, 1975, 2280, 2923, 3301, 3308, 3309, 3312, 3320, 4259, 4738, 5034, 5478, 5479, 5813, 5860, 6124, 6128, 6134, 6137, 6159, 6189, 6203, 6217, 6222, 6233, 6238, 7184, 7203, 7295, 7317, 7334, 7336, 7345, 7415, 8078, 8745, 9520, 9829, 10130, 10134, 10574, 10575, 10576, 10598, 10694, 10808, 10963, 22824, 23052, 23191, 23193, 25813, 26278, 51559, 53616, 55611, 55690, 55735, 55832, 85365, 150726, 259217, 360200 |
| **CELL METABOLISM**<br># genes: 57<br>LIDs: 291, 292, 348, 760, 1622, 2023, 2026, 2180, 2181, 2194, 2597, 2710, 2739, 2805, 2819, 2947, 2949, 2950, 3098, 3295, 3417, 3939, 4035, 4190, 4830, 4831, 5052, 5211, 5213, 5214, 5223, 5230, 5297, 5306, 5447, 5834, 6513, 6515, 6520, 6533, 6534, 6535, 7001, 7086, 8140, 8396, 9615, 10991, 11343, 23396, 26227, 51097, 55276, 55750, 55754, 79837, 340146 |
| **UNKNOWN**<br># genes: 58<br>LIDs: 308, 309, 310, 311, 320, 321, 351, 1855, 2319, 2596, 2631, 3916, 3920, 5621, 6252, 6305, 6622, 7466, 8508, 8537, 9501, 9546, 9758, 9764, 9796, 10211, 10313, 10439, 10513, 10550, 10787, 10972, 23154, 23312, 23335, 23504, 26090, 26960, 27095, 57142, 57552, 58485, 65125, 78997, 80863, 84152, 84293, 85300, 114569, 126003, 157378, 285368, 286205, 343990, 347730, 387104, 388336, 389813 |
| ***Pathways*** |
| **METABOTROPIC GLUTAMATE RECEPTOR**<br># genes: 65<br>LIDs: 774, 777, 781, 782, 784, 785, 2596, 2767, 2776, 2782, 2783, 2784, 2785, 2786, 2787, 2788, 2790, 2791, 2911, 3845, 4082, 4905, 5331, 5332, 5578, 5579, 5580, 5581, 5582, 5594, 5595, 5604, 5605, 5894, 6505, 6509, 6511, 6512, 6616, 6714, 6812, 6813, 6814, 6853, 6857, 9254, 9630, 10368, 10369, 10497, 10681, 23025, 23236, 27091, 27092, 54331, 55799, 55970, 57030, 57084, 59345, 93589, 148281, 246213, 440279 |
| **CANABINOID**<br># genes: 86<br>LIDs: 107, 108, 109, 111, 114, 673, 773, 774, 777, 781, 782, 784, 785, 1268, 1269, 2596, 2770, 2771, 2773, 2782, 2783, 2784, 2785, 2786, 2787, 2788, 2790, 2791, 3760, 3762, 3763, 3765, 3845, 4082, 4905, 5331, 5332, 5573, 5575, 5576, 5577, 5578, 5579, 5580, 5581, 5582, 5594, 5595, 5604, 5605, 5609, 5894, 5909, 6616, 6812, 6813, 6814, 6853, 6854, 6857, 8224, 8775, 9254, 9751, 10368, 10369, 10411, 10497, 10681, 11069, 11343, 22895, 22999, 23025, 23236, 23557, 27091, 27092, 54331, 55799, 55970, 59345, 93589, 148281, 196883, 440279 |

| |
|---|
| **DOPAMINE**<br># genes: 73<br>LIDs: 107, 108, 109, 111, 114, 673, 773, 774, 777, 781, 782, 784, 785, 1312, 1644, 1812, 1816, 2596, 2778, 2782, 2783, 2784, 2785, 2786, 2787, 2788, 2790, 2791, 3845, 4082, 4128, 4129, 4905, 5573, 5575, 5576, 5577, 5595, 5604, 5605, 5609, 5894, 6531, 6616, 6812, 6813, 6814, 6853, 6854, 6857, 7054, 8224, 8775, 9254, 9751, 10368, 10369, 10411, 10681, 11069, 22820, 22895, 22999, 23557, 27091, 27092, 54331, 55799, 55970, 59345, 93589, 148281, 196883 |
| **SEROTONIN**<br># genes: 111<br>LIDs: 107, 108, 109, 111, 114, 673, 773, 774, 775, 776, 777, 778, 781, 782, 784, 785, 801, 805, 808, 810, 814, 815, 816, 817, 818, 1644, 2596, 2770, 2771, 2773, 2782, 2783, 2784, 2785, 2786, 2787, 2788, 2790, 2791, 3350, 3351, 3352, 3760, 3762, 3763, 3765, 3845, 4082, 4128, 4129, 4905, 5331, 5332, 5573, 5575, 5576, 5577, 5578, 5579, 5580, 5581, 5582, 5594, 5595, 5604, 5605, 5609, 5894, 5909, 6532, 6616, 6812, 6813, 6814, 6843, 6853, 6854, 6855, 6857, 7166, 8224, 8536, 8775, 9254, 9751, 10368, 10369, 10411, 10497, 10681, 11069, 22895, 22999, 23025, 23236, 23557, 27091, 27092, 54331, 55450, 55799, 55970, 57118, 57172, 59345, 91860, 93589, 121278, 148281, 196883, 440279 |

**Table S2. Main References Used to Identify Functional Synaptic Groups and Canonical Pathways**

| |
|---|
| 1: Emes, RD, Pocklington AJ, Anderson CN, Bayes A, Collins MO, Vickers CA, Croning MD, Malik BR, Choudhary JS, Armstrong JD, Grant SG. Evolutionary expansion and anatomical specialization of synapse proteome complexity. Nat Neurosci. 2008 Jul;11(7):799-806. Epub 2008 Jun 8. PubMed PMID: 18536710. |
| 2: Collins MO, Husi H, Yu L, Brandon JM, Anderson CN, Blackstock WP, Choudhary JS, Grant SG. Molecular characterization and comparison of the components and multiprotein complexes in the postsynaptic proteome. J Neurochem. 2006 Apr;97 Suppl 1:16-23. PubMed PMID: 16635246. |
| 3: Cheng D, Hoogenraad CC, Rush J, Ramm E, Schlager MA, Duong DM, Xu P, Wijayawardana SR, Hanfelt J, Nakagawa T, Sheng M, Peng J. Relative and absolute quantification of postsynaptic density proteome isolated from rat forebrain and cerebellum. Mol Cell Proteomics. 2006 Jun;5(6):1158-70. Epub 2006 Feb 28. PubMed PMID: 16507876. |
| 4: Peng J, Kim MJ, Cheng D, Duong DM, Gygi SP, Sheng M. Semiquantitative proteomic analysis of rat forebrain postsynaptic density fractions by mass spectrometry. J Biol Chem. 2004 May 14;279(20):21003-11. Epub 2004 Mar 12. PubMed PMID: 15020595. |
| 5: Fang X, Lee CS. Proteome characterization of mouse brain mitochondria using electrospray ionization tandem mass spectrometry. Methods Enzymol. 2009;457:49-62. PubMed PMID: 19426861. |
| 6: Morciano M, Beckhaus T, Karas M, Zimmermann H, Volknandt W. The proteome of the presynaptic active zone: from docked synaptic vesicles to adhesion molecules and maxi-channels. J Neurochem. 2009 Feb;108(3):662-75. PubMed PMID: 19187093. |
| 7: Li KW, Miller S, Klychnikov O, Loos M, Stahl-Zeng J, Spijker S, Mayford M, Smit AB. Quantitative proteomics and protein network analysis of hippocampal synapses of CaMKIIalpha mutant mice. J Proteome Res. 2007 Aug;6(8):3127-33. Epub 2007 Jul 11. PubMed PMID: 17625814. |
| 8: Schrimpf SP, Meskenaite V, Brunner E, Rutishauser D, Walther P, Eng J, Aebersold R, Sonderegger P. Proteomic analysis of synaptosomes using isotope-coded affinity tags and mass spectrometry. Proteomics. 2005 Jul;5(10):2531-41. PubMed PMID: 15984043. |
| 9: Li KW, Hornshaw MP, Van Der Schors RC, Watson R, Tate S, Casetta B, Jimenez CR, Gouwenberg Y, Gundelfinger ED, Smalla KH, Smit AB. Proteomics analysis of rat brain postsynaptic density. Implications of the diverse protein functional groups for the integration of synaptic physiology. J Biol Chem. 2004 Jan 9;279(2):987-1002. Epub 2003 Oct 7. PubMed PMID: 14532281. |
| 10: Abul-Husn NS, Bushlin I, Morón JA, Jenkins SL, Dolios G, Wang R, Iyengar R, Ma'ayan A, Devi LA. Systems approach to explore components and interactions in the presynapse. Proteomics. 2009 Jun;9(12):3303-15. PubMed PMID: 19562802; PubMed Central PMCID: PMC2766278. |
| 11: Takamori S, Holt M, Stenius K, Lemke EA, Grønborg M, Riedel D, Urlaub |

H,Schenck S, Brügger B, Ringler P, Müller SA, Rammner B, Gräter F, Hub JS, De GrootBL, Mieskes G, Moriyama Y, Klingauf J, Grubmüller H, Heuser J, Wieland F, Jahn R. Molecular anatomy of a trafficking organelle. Cell. 2006 Nov 17;127(4):831-46. PubMed PMID: 17110340.

12: de Jong AP, Verhage M. Presynaptic signal transduction pathways that modulate synaptic transmission. Curr Opin Neurobiol. 2009 Jun;19(3):245-53. Epub 2009 Jun 24. Review. PubMed PMID: 19559598.

13: Dresbach T, Nawrotzki R, Kremer T, Schumacher S, Quinones D, Kluska M, Kuhse J, Kirsch J. Molecular architecture of glycinergic synapses. Histochem Cell Biol. 2008 Oct;130(4):617-33. Epub 2008 Aug 22. Review. PubMed PMID: 18719933.

14: Raiteri M. Presynaptic metabotropic glutamate and GABAB receptors. Handb Exp Pharmacol. 2008;(184):373-407. Review. PubMed PMID: 18064420.

15: Brown DA, Sihra TS. Presynaptic signaling by heterotrimeric G-proteins. Handb Exp Pharmacol. 2008;(184):207-60. Review. PubMed PMID: 18064416.

16: Lang T, Jahn R. Core proteins of the secretory machinery. Handb Exp Pharmacol. 2008;(184):107-27. Review. PubMed PMID: 18064413.

17: Bai F, Witzmann FA. Synaptosome proteomics. Subcell Biochem. 2007;43:77-98. Review. PubMed PMID: 17953392.

18: Khanna R, Li Q, Bewersdorf J, Stanley EF. The presynaptic CaV2.2 channel-transmitter release site core complex. Eur J Neurosci. 2007 Aug;26(3):547-59. PubMed PMID: 17686036.

19: Khanna R, Zougman A, Stanley EF. A proteomic screen for presynaptic terminal N-type calcium channel (CaV2.2) binding partners. J Biochem Mol Biol. 2007 May 31;40(3):302-14. PubMed PMID: 17562281.
